# Potential impact on prevalence of obesity in the UK of a 20% price increase in high sugar snacks: modelling study

**DOI:** 10.1136/bmj.l4786

**Published:** 2019-09-04

**Authors:** Pauline F D Scheelbeek, Laura Cornelsen, Theresa M Marteau, Susan A Jebb, Richard D Smith

**Affiliations:** 1 Department of Population Health, London School of Hygiene and Tropical Medicine, London WC1E 7HT, UK; 2 Centre on Climate Change and Planetary Health, London School of Hygiene and Tropical Medicine, London, UK; 3 Department of Public Health, Environments and Society, London School of Hygiene and Tropical Medicine, London, UK; 4 Behaviour and Health Research Unit, Institute of Public Health, University of Cambridge, Cambridge, UK; 5 Nuffield Department of Primary Care Health Sciences, University of Oxford, Oxford, UK; 6 College of Medicine and Health, University of Exeter, Exeter, UK

## Abstract

**Objective:**

To estimate the potential impact on body mass index (BMI) and prevalence of obesity of a 20% price increase in high sugar snacks.

**Design:**

Modelling study.

**Setting:**

General adult population of the United Kingdom.

**Participants:**

36 324 households with data on product level household expenditure from UK Kantar FMCG (fast moving consumer goods) panel for January 2012 to December 2013. Data were used to estimate changes in energy (kcal, 1 kcal=4.18 kJ=0.00418 MJ) purchase associated with a 20% price increase in high sugar snacks. Data for 2544 adults from waves 5 to 8 of the National Diet and Nutrition Survey (2012-16) were used to estimate resulting changes in BMI and prevalence of obesity.

**Main outcome measures:**

The effect on per person take home energy purchases of a 20% price increase for three categories of high sugar snacks: confectionery (including chocolate), biscuits, and cakes. Health outcomes resulting from the price increase were measured as changes in weight, BMI (not overweight (BMI <25), overweight (BMI ≥25 and <30), and obese (BMI ≥30)), and prevalence of obesity. Results were stratified by household income and BMI.

**Results:**

For income groups combined, the average reduction in energy consumption for a 20% price increase in high sugar snacks was estimated to be 8.9×10^3^ kcal (95% confidence interval −13.1×10^3^ to −4.2×10^3^ kcal). Using a static weight loss model, BMI was estimated to decrease by 0.53 (95% confidence interval −1.01 to −0.06) on average across all categories and income groups. This change could reduce the UK prevalence of obesity by 2.7 percentage points (95% confidence interval −3.7 to −1.7 percentage points) after one year. The impact of a 20% price increase in high sugar snacks on energy purchase was largest in low income households classified as obese and smallest in high income households classified as not overweight.

**Conclusions:**

Increasing the price of high sugar snacks by 20% could reduce energy intake, BMI, and prevalence of obesity. This finding was in a UK context and was double that modelled for a similar price increase in sugar sweetened beverages.

## Introduction

Over the past decades the prevalence of obesity has increased steeply, with rates tripling globally between 1975 and 2016. In 2016, about two billion adults (aged 18 or more) worldwide were overweight, of whom more than 650 million were classified as obese.^1^ Obesity is a major risk factor for several chronic conditions, including ischaemic heart disease, stroke, many cancers, and type 2 diabetes.^2^ In the UK, the prevalence of obesity among adults was estimated at 27.8% (95% confidence interval 24.9% to 30.7%) in 2016,^3^ higher than the average of 19.5% reported by the Organisation for Economic Co-operation and Development.^4^ However, noticeable differences exist in the prevalence of obesity in relation to deprivation and income.^5^ ^6^ ^7^ In 2016, 38% of women living in the most deprived areas in England were classified as obese compared with 20% living in the least deprived areas.^8^ Among children aged 2-15 years, 26% of those living in households in the lowest income fifth were classified as obese or overweight compared with 18% of children in households in the highest fifth.^9^

High levels of dietary free sugars increase the risk of obesity and diabetes.^10^ ^11^ Sugar sweetened beverages often make up a substantial part of consumed free sugars,^12^ and they have been a major focus of policy.^13^ In the UK, however, high sugar snacks, such as confectionery, cakes, and biscuits make a greater contribution to intakes of free sugars as well as energy than sugar sweetened beverages.^14^ The National Diet and Nutrition Survey (NDNS) shows that on average sugar sweetened beverages contribute 2% of total energy and 11% of free sugar intake compared with 12% of total energy and 26% of free sugar intake from biscuits, cakes, and confectionery combined.^13^ Reducing purchases of high sugar snacks therefore has the potential to make a greater impact on population health than that achieved by reducing the purchase of sugar sweetened beverages.

Health related taxes have been recommended by the World Health Organization and a recent task force on fiscal policies for health to reduce purchases of sugar sweetened beverages.^15^ ^16^ Early adopters of such a taxation strategy include Mexico (2014), Hungary (2012), and Finland (2011).^17^ ^18^ These countries introduced taxes not only on sugar sweetened beverages but also on other unhealthy foods, including high sugar snacks. In Mexico, for example, all “non-essential foods” with 275 or more kcal/100 g are taxed at 8%, including biscuits and cereal bars.^19^ In Hungary, prepacked high sugar sweets with more than 25 g of sugar are taxed at 130 HUF (£0.40; €0.40; $0.40) per kilogram. Finland had a tax on sweets and ice cream (about 75p per kilogram) between 2011 and 2017. Existing evaluations suggest that the tax in Hungary, which also applied to products high in salt, reduced purchases of the taxed foods by 3.4%.^20^ In Mexico the tax on non-essential foods was estimated to have reduced purchases by 5-6%, with greater effects (reduction by 12.3%) among those with higher baseline purchases of taxed foods.^21^ ^22^

In the UK, the Soft Drinks Industry Levy (SDIL) came into effect in April 2018, with an 18p and 24p tax per litre on drinks containing 5 or more and 8 or more grams of sugar per 100 millilitres, respectively.^23^ The two tiered nature of the levy has encouraged a wave of reformulation by the beverage industry, with at least 50% of manufacturers reducing the sugar content of their products.^23^ Since 2016, a voluntary sugar reduction and reformulation programme has also covered 10 categories of high sugar foods that contribute most to the sugar intakes of children, which challenged producers to reduce the amount of sugar by 20% by 2020.^13^ After the first year, an overall 2% reduction took place compared with the interim target of 5%, with particularly small reductions in confectionery. For example, between 2015 and 2017 there was only a 1% reduction in sugar content of confectionery compared with a 5-6% decline in the sugar content of yoghurt and breakfast cereals.^24^

Given the modest impacts of the voluntary sugar reduction programme relative to the reductions seen through reformulation of sugar sweetened beverages, where a levy is also applied, we estimated the potential impact of a price increase in high sugar snacks on total energy purchase and subsequent health. We examined these likely effects by both body mass index (BMI) and household income group to assess the potential of price increases in high sugar snacks to reduce their purchase among those with the highest BMI and therefore at greatest risk of related diseases, and to assess their potential to reduce the health inequalities arising from higher rates of obesity among those in lower income groups.

## Methods

**Fig 1:**
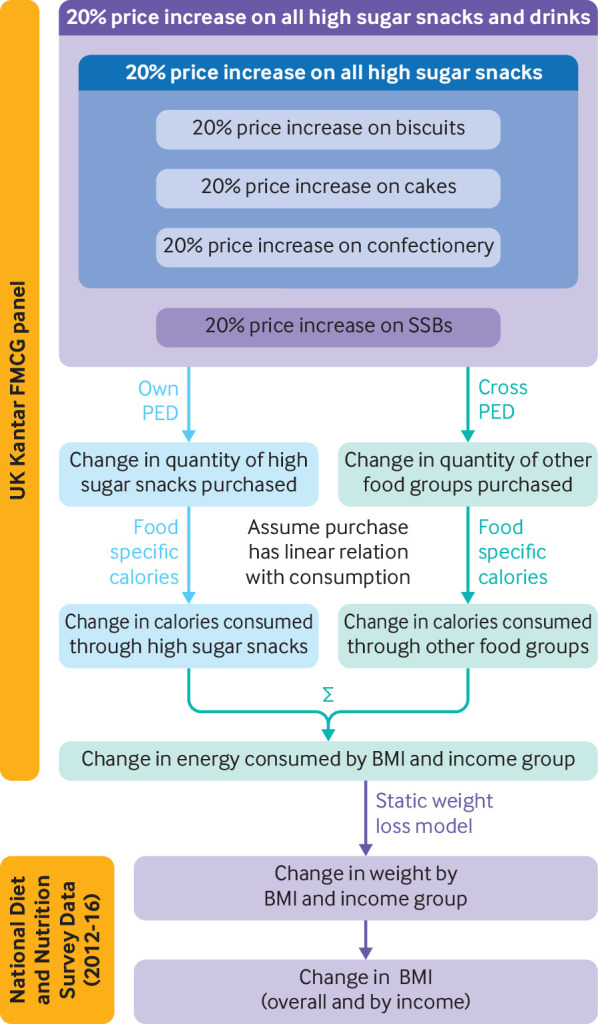
Modelled causal pathway between price increases in high sugar snacks and changes in weight and obesity. PED=price elasticity of demand; BMI=body mass index; FMCG=fast moving consumer goods; SSBs=sugar sweetened beverages

We modelled the effect of a 20% price increase in high sugar snacks on the energy purchased and used this to estimate changes in weight and prevalence of overweight (BMI ≥25 and <30) and obesity (BMI >30) in the UK. We used a 20% price change, based on tax scenarios for sugar sweetened beverages in the modelling literature,^25^ ^26^ and presumed that this would lead to an equivalent change in prices of high sugar snacks (fig 1).

Data on household expenditure at product level were taken from the UK Kantar FMCG (fast moving consumer goods) panel (n=36 324 households) for January 2012 to December 2013.^27^ The household sample is representative of the population in Great Britain for household size, number of children, social class, geographical region, and age of the main shopper. These data have been used in numerous studies in the UK to understand patterns of food demand (eg,^28^ ^29^) and have been shown to compare well to the Living Cost and Food Survey, which is the official government data collection on household expenditures.^30^ ^31^ Participants are recruited by Kantar to the open panel through post and e-mail, and Kantar assess panel representativeness at intervals of four weeks. Households supply data on items purchased and brought home by scanning barcodes of the products and sending in digital images of cash register receipts. Households are additionally supplied with barcodes to record purchases of unpackaged products, such as fruit and vegetables. The dataset includes information on purchases (volume and expenditure) of food and drinks from a wide range of outlets, including large retailers, supermarkets, butchers, greengrocers, and corner shops. The data exclude all purchases of food and beverages not brought home (eg, consumed away from home). Explicit comparison and validation studies of Kantar FMCG data in comparison to the Living Cost and Food Survey are available for earlier years (2003-09) and show a good fit. Furthermore, the use of data on commercial food purchases for public health research is growing and has been deemed a valuable tool for nutrition research.^32^

As in a previous study^33^ we aggregated data from all foods and beverages into 13 distinct groups. As high sugar snacks were the focus of this study, we included three categories, defined as high sugar products at ambient temperature—typically to be consumed on the go without utensils. These were confectionery (including chocolate), biscuits, and cake (piece, slice, or portion). The last group did not include larger cakes (meant for sharing) and proved to be the least straightforward category to assign.

The impact of price changes on the demand for a product is the price elasticity of demand (PED). This indicates the change in demand—expressed as a percentage—given a 1% change in price. It is divided into “own PED,” showing the change in demand for a certain product if its own price changed, and “cross PED” showing the change in demand for a certain product if the price of other products changed. For this study, we calculated own PED and cross PED by income and BMI of the main household purchaser using an adapted version of the Almost Ideal Demand System, which is described in detail elsewhere.^33^ The total sample size was 36 324 for estimating own PED and cross PED by income and 27 115 by household income and BMI group (this is different from the sample sizes in the energy purchase analysis). Group specific own PED are reported in appendix table B, and cross PEDs can be found in the technical appendix.

Data on the energy content of the foods and beverages purchased are recorded twice a year by Kantar from products available in the market. Average baseline daily purchases of energy were estimated using 2013 data only (n=32 620 households) and calculated as per household member per day, without adjustment for age or sex. Baseline purchases were expressed in weighted means to account for sampling selection of the panel and underreporting using pooled weights provided with the data.

### Statistical analyses

Analyses were carried out on the full sample and subsamples by annual income (low <£20 000, middle £20 000–49 999, and high ≥£50 000) and BMI (not overweight (BMI <25), overweight (25 to <30), and obese (≥30)) of the main household purchaser—that is, the person generally responsible for food purchase in a household. As not all participants on the 2013 database reported income (11% missing, 3554 households), the final estimation sample used to estimate changes included 29 066 households. BMI data were missing from a further 13% (4290 households), hence the number of participants for the stratified analysis by BMI group equalled 24 776.

We used the own PED and cross PED matrix for all BMI and income groups to estimate change in per person energy purchase simulating a 20% price increase in one or multiple high sugar snack groups. New energy purchase totals per income or BMI group, or both were constructed by totalling scenario specific energy purchases for each food group. Confidence intervals of changes in energy purchase were constructed using intragroup variability of energy purchase as well as variance of estimates for own PEDs and cross PEDs (see equations 1 and 2 in the technical appendix).

To estimate the combined effect of multiple price increases on energy purchase we used a chained version of the methods previously described. This covered the combined effect of a 20% price increase in confectionery, biscuits, and cake.

We used the change in average energy purchased per household member (unadjusted for age or sex) as a proxy for change in energy intake of the main purchaser to estimate change in weight. A static model estimated average weight change for each BMI and income group (see equation 3 in the technical appendix) based on change in energy intake. An average of 7715 kcal/kg (1 kcal=4.18 kJ=0.00418 MJ) weight loss was used, with a standard deviation of 245 kcal (representing a 7% coefficient of variation and assumed a normal distribution, in line with values reported in the literature for energy expenditure in healthy adults (eg,^34^ ^35^). We disregarded any changes in energy expenditure resulting from weight fluctuations, as we expected the change in weight and hence energy expenditure to be small over the course of one year. For each subgroup we ran 1000 Monte Carlo simulations using best estimates, variance of change in energy purchase and variance of energy expenditure.

The derived weight loss was used to estimate changes in BMI and prevalence of overweight and obesity in adults (≥16 years) based on data from the National Diet and Nutrition Survey (NDNS) waves 5 to 8 (2012-16; n=3145 adults^36^). Data on household income were missing for 447 (14.2%) adults and for BMI in a further 154 (4.9%), hence analyses were performed on 2544 adults. The NDNS is a nationally representative, rolling cross sectional survey designed to “collect detailed, quantitative information on the food consumption, nutrient intake and nutritional status of the general population.”^36^ A trained data collector measures height and weight of participants and therefore data are less prone to (non-differential) misclassification than self report. Data on consumption of high sugar snacks were also available in NDNS. As these were self reported quantities—and therefore potentially prone to non-differential underreporting—in our view the data from the Kantar FMCG panel are more accurate for our baseline energy purchase data in the previous steps described. Body weight for each NDNS participant was projected by subgroup, running 1000 Monte Carlo simulations using best estimates and variance of weight loss and BMI. Details of the uncertainty approach applied can be found in the technical appendix.

Data from the Kantar FMCG panel include households with and without children, with food purchases registered at household level. The proportional share of purchased foods that is consumed by children in the household could vary greatly, with potential to impact on the results of the models. We therefore performed sensitivity analyses for several steps of the model whereby households with one or more children were excluded.

### Patient and public involvement

The public was not involved in the design, execution, and interpretation of this study. All data used in this study are, however, nationally representative and obtained through public panels (Kantar) and samples of the public (NDNS).

## Results

Supplementary appendix 1 provides descriptive characteristics of the sample population, body mass index (BMI), income specific own price elasticity of demands (PEDs) and cross PEDs, and baseline purchases of energy (for each household member per day).

Overall, the own PED ranged between −0.29 and −1.06, with high income households showing generally least price sensitive demand across foods and beverages. Households classified as obese or overweight were less price responsive than those classified as normal weight, except for two food groups (confectionery and “other” drinks (eg, juice)). Baseline energy purchases ranged between 1808 and 2406 kcal (1 kcal=4.18 kJ=0.00418 MJ) per day for each household member. The baseline energy purchases were highest for low income households and lowest for high income households. In each income group, households classified as obese purchased most energy.

Table 1 shows the estimated changes per year in purchases of energy by household income group, based on a 20% price increase in each of the three categories of high sugar snacks, as well as the combined effect.

**Table 1:** Change in annual energy purchase by household income category and 20% price increase scenario

Household income (£)	No of participants	Change in annual energy (kcal) purchase (95% CI) with 20% price increase			
		Biscuits	Confectionery*	Cake†	High sugar snacks
Low (<20 000)	10 308	−3709 (−4401 to −3016)	−4805 (−5326 to −4284)	1052 (1316 to 789)	−6855 (−10 034 to −3675)
Middle (20 000-49 999)	14 332	−4395 (−5011 to −3780)	−3319 (−3622 to −3015)	−3073 (−3745 to −2401)	−10 469 (−14 711 to −6227)
High (≥50 000)	4426	−1640 (−2126 to −1154)	−3230 (−3797 to −2662)	−4182 (−5860 to −2403)	−9001 (−16 039 to −1962)
All	29 066	−3732 (−4409 to −3056)	−3832 (−4257 to −3407)	−1779 (−2296 to −1262)	−8964 (−13 772 to −4155)

1 kcal=4.18 kJ=0.00418 MJ. £1.00=$1.2; €1.1.

* Includes chocolate.

† Piece, slice, or portion.

A 20% price increase in confectionery and biscuits, or both reduced energy purchased across all income groups. The analysis on confectionery showed a more prominent reduction in energy purchase among low income households compared with middle and high income households. A 20% price increase in biscuits showed a less prominent reduction in energy purchase among high income households compared with low and middle income households. Results for cakes were mixed: among low income households, a 20% price increase led to a small increase in energy purchases primarily driven by the substitution effect towards other foods. For income groups combined, the average change in energy consumption for a price increase in high sugar snacks was −8.9×10^3^ kcal (95% confidence interval −13.1×10^3^ to −4.2×10^3^ kcal). Sensitivity analysis revealed that the modelled impact of price changes on energy purchase was marginally more profound in households without children, especially low income households. Differences were, however, not statistically significant (see supplementary appendix 2).

**Fig 2:**
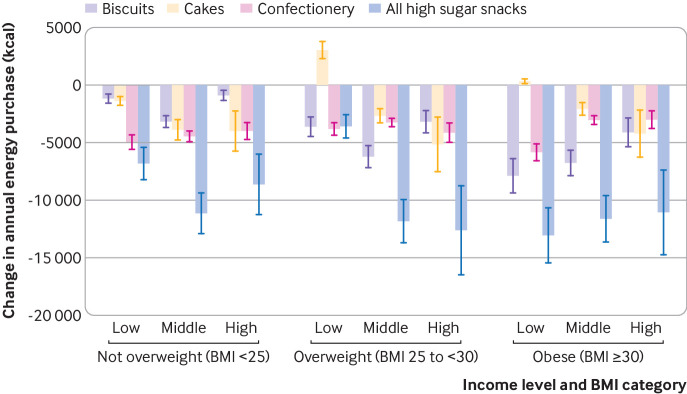
Impact of 20% price increase in high sugar snacks on change in annual energy purchase by body mass index (BMI) and household income. Stratification by household income (low <£20 000, middle £20 000-49 999, high ≥£50 000) and BMI. 1 kcal=4.18 kJ=0.00418 MJ). £1.00=$1.2; €1.1. Confectionery includes chocolate. Whiskers represent 95% confidence intervals

Figure 2 shows change in energy purchase of the further stratified analysis. using income and BMI specific own PEDs and cross PEDs. Price increases in each of the high sugar snack groups, and their combinations, were associated with a reduction overall in energy purchased among all BMI and income groups, with two exceptions that showed small increases (a price increase in cakes in households classified as low income and overweight and those classified as low income and obese). Among the three high sugar snack groups, price increases in biscuits showed the strongest reduction in energy purchased in the group classified as obese, with the greatest effects in low income households. Households classified as not being overweight appeared to be most responsive to price increases in confectionery (including chocolate), especially among low income households. The impact of price increases in various snack groups in the group classified as overweight showed no clear pattern.

Average annual change in energy purchase per person for those classified as obese, based on a 20% price increase in high sugar snacks, was −13.1×10^3^ kcal (95% confidence interval −15.5×10^3^ to −10.7×10^3^ kcal) for low income households, −11.6×10^3^ kcal (−13.6×10^3^ to −9.6×10^3^ kcal), for middle income households, and −11.1×10^3^ kcal (−14.8×10^3^ to −7.4×10^3^ kcal) for high income households (fig 2).

Among households classified as obese, the effect of price increases in biscuits and confectionery on energy purchase varied by income: those from low income households showed most responsive to price increases compared with those from middle and high income households, leading to relatively larger reductions in energy purchase in the low income households. Among households classified as overweight, similar reductions in energy purchased were observed in middle and high income households, whereas price increases in high sugar snacks in overweight low income households were associated with relatively small reductions in energy purchased.

Sensitivity analysis excluding households with children showed similar but slightly more profound effects of price increases on energy purchase—especially among obese low income households. The differences with the main model were not, however, statistically significant (see supplementary appendix 2). Analyses were repeated including a Bonferroni correction for multiple comparisons. Uncertainty around the estimates increased by about 16% on average, but all changes in energy purchase from high sugar snacks remained significant (see supplementary appendix 3).

**Fig 3:**
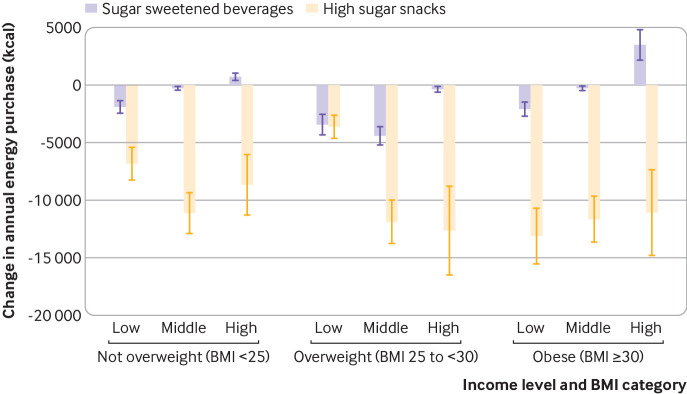
Impact of 20% price increase in sugar sweetened beverages and high sugar snacks on change in annual energy purchase by body mass index (BMI) and household income group. Stratification by income group (low <£20 000, middle £20 000-49 999, high ≥£50 000) and BMI group. £1.00=$1.2; €1.1. Whiskers represent 95% confidence intervals

Figure 3 compares the impact of two (independent) price increase scenarios (20% price increase in sugar sweetened beverages and 20% price increase in high sugar snacks) on annual energy purchase. The impact of price increases in high sugar snacks was substantially larger on energy purchased than an equivalent price increase in sugar sweetened beverages throughout all income and BMI groups. Following the scenario of a 20% price increase in sugar sweetened beverages, in households classified as obese the annual purchase of energy changed by −2.1×10^3^ kcal (−2.6 to −1.5×10^3^ kcal), −0.3×10^3^ kcal (−0.4 to −0.1×10^3^ kcal), and 3.5×10^3^ kcal (2.2 to 4.8×10^3^ kcal), for low, middle, and high income households, respectively. These are lower than the changes resulting from a 20% price increase in high sugar snacks.

Table 2 shows the results of modelled weight changes over one year by household income and BMI, based on changes in energy purchased, under the assumption that change in energy purchased is a proxy for change in energy consumption. A 20% price increase in high sugar snacks and sugar sweetened beverages was estimated to result in weight changes varying from −911 g (95% confidence interval −1115 to −707 g) in the overweight low income households to −2100 g (−2530 to −1671 g) in middle income overweight households one year after the introduction of the price increase.

**Table 2:** Estimated average weight change (g) after first year by 20% price increase, body mass index (BMI),* and household income†

BMI group by household income	No of participants	Change in weight (g) (95% CI)					
		Beverages	Biscuits	Confectionery‡	Cake	High sugar snacks	Beverages and snacks
Not overweight:							
Low	372	−247 (−316 to −178)	−152 (−189 to −115)	−645 (−748 to −543)	−180 (−229 to −129)	−889 (−1340 to −439)	−1136 (−1387 to −885)
Middle	473	−35.7 (−74.9 to 3.4)	−419 (−501 to −336)	−578 (−661 to −495)	−505 (−644 to −366)	−1451 (−2123 to −780)	−1494 (−1801 to −1188)
High	191	97.5 (3.0 to 192)	−115 (−211 to −8.4)	−521 (−631 to −411)	−524 (−773 to −276)	−1126 (−2057 to −195)	−1010 (−1343 to −678)
Overweight:							
Low	273	−445 (−570 to −321)	−472 (−582 to −363)	−495 (−575 to −415)	+395 (287 to 504)	−473 (−717 to −229)	−911 (−1115 to −707)
Middle	440	−574 (−702 to −445)	−809 (−967 to −651)	−423 (−483 to −362)	−346 (−442 to −250)	−1544 (−2259 to −830)	−-2100 (−2530 to −1671)
High	156	−48.1 (−70.1 to 0.8)	−418 (−543 to −293)	−539 (−653 to −424)	−665 (−981 to −349)	−1646 (−3005 to −287)	−1726 (−2291 to −1162)
Obese:							
Low	266	−270 (−346 to −194)	−1023 (−1254 to −793)	−762 (−884 to −640)	41.4 (−21.6 to 104.3)	−1706 (−2568 to −844)	−1987 (−2423 to −1550)
Middle	292	−32.3 (−141.6 to 76.9)	−879 (−1051 to −706)	−399 (−457 to −340)	−270 (−345 to −194)	−1512 (−2213 to −810)	−1604 (−1935 to −1274)
High	81	457 (298 to 616)	−531 (−690 to −371)	−393 (−479 to −307)	−551 (−815 to −288)	−1448 (−2648 to −247)	−1024 (−1365 to −684)
All	2544	−203 (−269 to −137)	−550 (−691 to −409)	−538 (−633 to −444)	−262 (−361 to −162)	−1301 (−2088 to −513)	−1511 (−1898 to −1121)

* Not overweight (BMI <25), overweight (25 to <30), and obese (≥30).

† Low <£20 000, middle £20 000-49 999, high ≥£50 000.

‡ Includes chocolate.

Pooling all BMI and income groups, a price increase of high sugar snacks and beverages combined is estimated to decrease BMI on average by −0.53 (95% confidence interval −1.01 to −0.06). In households classified as obese this ranged from −0.36 (−0.63 to −0.09) for high income to −0.72 ( −1.03 to −0.41) for low income households a year after the introduction of the price increase (see supplementary appendix 4).

A positive weight gain was estimated for a 20% price increase in sugar sweetened beverages among high income households classified as obese. This could be explained by substitution towards high sugar snacks with higher energy content (see supplementary appendix). For households in the same income category but classified as not overweight or overweight the impact of a price increase in sugar sweetened beverages on weight change was marginal to statistically and clinically non-significant.

The projection of the estimated weight changes on a representative sample of the UK population shows a noticeable shift in the population distribution of BMI, with a decrease in the proportion classified as obese (BMI ≥30) and an increase in the proportion classified as not overweight (BMI <25). The simulated decreases in the prevalence of obesity are most marked in low and middle income households. Based on a 20% price increase in high sugar snacks, the prevalence of obesity after one year would decrease by 3.1 percentage points (95% confidence interval −3.6 to −2.6 percentage points) in low income households, 2.5 percentage points (−2.9 to −2.1 percentage points) in middle income households, and 2.3 percentage points (−3.2 to −1.5 percentage points) in high income households. For the UK population this would result in a reduction of 2.68 percentage points (−3.7 to −1.7 percentage points) in the prevalence of obesity (see supplementary appendix 5).

## Discussion

A 20% price increase in high sugar snacks has the potential to reduce overall energy purchased among all body mass index (BMI) and household income groups in the United Kingdom. The estimated population level reduction in prevalence of obesity in the first year was 2.7 percentage points.

Furthermore, our results suggest that a 20% tax on high sugar snacks could also contribute to reducing health inequalities driven by diet related disease, given the potential for the greatest reduction in sugar consumption in households classified as obese (BMI ≥30) and low income (<£20 000 annually). In low income households classified as obese and middle income households classified as overweight, weight change based on a price increase in high sugar snacks and sugar sweetened beverages combined was estimated to be about 2 kg after 12 months. The estimated impacts were smaller in middle and high income households classified as obese, which can partly be explained by the differences in own price elasticity of demands (PEDs) and cross PEDs and also by the greater volume of high sugar snacks and beverages purchased in low income households classified as obese.

Our analysis followed an energy intake “pathway” to estimate the impact of a 20% price increase and subsequent reduced purchase of high sugar snacks and beverages (as a proxy for consumption) on body weight. In addition to this pathway, there could be other, direct adverse health impacts of high sugar intake, with reported increased risks of diabetes and several cardiovascular diseases.^37^ Reducing the purchase of high sugar snacks therefore might have additional benefits to population health than the ones assessed in this study.

We used a 20% price increase in a group of high sugar snacks to reflect the broad approach that has been undertaken for tax scenarios concerning sugar sweetened beverages in the modelling literature.^25^ ^26^ An alternative is to consider a tax related to nutrients,^38^ which in this case could be sugar or total energy. Most recent government proposals of health related food policies do use a nutrient profile model, which also takes into account “positive” contributions, such as fruit and vegetable or fibre content of the product. However, this generates increased complexity in both policy and analysis, and although this should be explored in future research for our purposes, the 20% price increase seemed most appropriate.

### Research in context

An observational study evaluating the (short term) impact of an 8% tax on energy dense, nutrient poor foods (including high sugar snacks) introduced in Mexico in 2014, concluded that the tax statistically significantly reduced the purchase of these foods in urban areas: reductions up to 15% were reported for southern Mexico.^39^ The tax intervention covered a much larger variety of foods than in our study, whereas the tax rate was lower, making it difficult to compare results. The authors concluded, however, that the strategy generated substantial revenue that could be used to finance policies for the treatment and prevention of obesity^39^: a 20% price increase in high sugar snacks could generate similar opportunities for obesity control in the UK.

Evidence from studies directly evaluating the economic and health impact of sugar taxation strategies was sparse. Although the taxation on sugar sweetened beverages was first introduced in the early 2010s, the lagged impact of dietary interventions limits the possibilities of accurately assessing the health impacts and contribution to the reduction of health inequalities in observational study designs. Although modelling studies project these to be favourable for lower socioeconomic groups,^40^ ^41^ ^42^ the results of observational studies remain inconclusive; specifically for sugar taxation.^43^ ^44^ It could be hypothesised that taxation strategies targeting non-essential foods that are high in sugar but have otherwise low additional nutritional value would have low regressive financial effects, similar to those of other non-essential products, such as sugar sweetened beverages, or even alcohol and tobacco.^44^

Our findings on daily calorie reductions from sugar sweetened beverages concur with previous results for a similar price increase,^45^ but they are much lower than that study’s estimates based on larger price increases of 18-75%.^26^

The estimated reduction of 0.53 in BMI with a 20% price increase is relatively large compared with other population level interventions aimed at weight reduction. For example, a recent study on active travel to work in the UK found that the BMI of people transitioning from car to active commuting decreased by 0.30 (95% confidence interval −0.47 to −0.13) after 4.4 years of follow-up.^46^ A systematic review and meta-analysis found a similar reduction, of 0.71 (−1.19 to−0.23), when the impact of walking group interventions was evaluated.^47^

Price increases in cakes were associated with increased energy purchased in two low incomes groups—those of households classified as overweight and those of households classified as obese. This could likely be explained by substitution towards more energy dense alternatives for cakes: a daily reduction of about 3-6 kcal from cakes was matched with an increase of about 16-18 kcal from substitutes (see the technical appendix). A similar increase in energy purchased was found for households classified as high income and obese. This could be related to positive cross PED towards the “other” group (aggregate of all remaining foods not allocated to a specific food group): a group providing nearly half of the daily energy intake.

In this UK based study we found a relatively high impact on overall energy purchased, and subsequent weight changes, of a 20% price increase in high sugar snacks versus sugar sweetened beverages. These results might also apply to other high income countries with similar consumption patterns of these two food groups, such as Australia, but might not align with countries with a much higher consumption of sugar sweetened beverages or a higher drinks to snacks ratio—such as the US or Mexico, where the effect of price increases in high sugar snacks might be less noticeable^48^ (see supplementary appendix 6). Regardless of the beverage versus snack purchase ratio, joint policies are expected to be approximately additive.

### Strengths and limitations of this study

The current study has several strengths. We modelled the effect of a price increase across a range of high sugar snacks. We also obtained information from several high quality databases comprising primary data on food purchase, food intake, and BMI of a representative national sample of the UK population. Data from the Kantar FMCG (fast moving consumer goods) panel, on which we based our predicted elasticity estimates and baseline purchases across the food groups, has been shown to produce similar estimates of budget and budget shares, compared with the Living Cost and Food Survey, the official, UK government collected data for household expenditures.^30^ ^31^

This study has a few limitations. Firstly, we excluded 25% of the households from analysis because of missing information on either income or BMI. It is unlikely that such missing information is related to price elasticity or purchase behaviour. As we present stratum specific data, missing information was expected to have little to no influence on the group specific estimates: however, it may have resulted in some bias for the pooled values across groups. Secondly, the baseline daily energy purchase estimates are sample average estimates that do not consider age or sex of the household members who could have different energy requirements. We were unable to adjust for these factors as we did not have information on these characteristics for household members. Thirdly, we used a static model for weight loss based on changes in energy consumption, which might not fully reflect actual mechanisms of weight change. However, the period considered in this study is one year, and modelled weight changes during that time were expected to be relatively small. Therefore changes in energy expenditure, as described previously, are expected to have marginal impacts on our results.^49^ Nonetheless, the model outcomes should be regarded as estimates on the order of scale of any such price increase strategy and would, as with any modelling study, require further “real life experience” testing to further quantify the effects on health. Fourthly, the study does not reflect on the substitution of nutrients alongside changes in energy. For example, reduction in energy from high sugar snacks could lead to substitution of other foods that are lower in energy content but perhaps higher in other nutrients of concern, such as saturated fats or salt. The health impacts of such substitutes should be further analysed and considered in the decision making process around food price policies. Furthermore, the satiety index of sugary snacks can vary greatly: some high sugar snacks could reduce overeating at meals, hence the overall impact of reduced consumption of high sugar snacks would be partly cancelled out by consumption of larger portions during mealtimes. Studies of sugary drinks only would be prone to this phenomenon, as the satiety effect of sugar sweetened beverages is generally low.^50^ Fifthly, we assumed that all food purchased was consumed, which is unlikely, and some food will inevitably be waste. However, although the link between purchasing and consumption is far from perfect, it is strong (eg,^51^), and our estimates on the effect of price rises on change in energy purchased is likely to be similar to that on consumption even if absolute values differ. The food items evaluated in this study represent take home purchases from retail outlets, such as supermarkets. Owing to lack of comparable data we did not include additional high sugar snacks purchased, for example, from cafés and restaurants and consumed on the go, and the consumption of these snacks as well as the effect of price increases might vary considerably across income groups. Our estimates of the overall effect are therefore conservative—albeit the implications on inequality are more difficult to assess a priori. Finally, we did not consider a specific pass through rate for tax (ie, the proportion of the price increase to be paid by the consumer) as found in studies evaluating food tax (eg^52^) and based on evidence around linear effects of price change on consumption—specifically for sugary beverages.^53^ As we specifically studied the effect of a 20% price increase to the consumer, it is less relevant whether this would be a true reflection of a particular pass through rate in the UK.

### Conclusions

Increasing the price of high sugar snacks by 20% could reduce energy intake and BMI to more than twice that observed for similar price increases on sugar sweetened beverages, but with strong variability across household income and BMI groups. Furthermore, evaluations of price increases on sugar sweetened beverages—– which faced similar analytical limitations to our study—have shown reductions in purchases closely aligned to the modelled predictions, suggesting price elasticities were reasonably valid. Although this might differ for high sugar snacks, it cannot be tested until such policies are implemented. Meanwhile this analysis provides policymakers with estimates of the relative magnitude of plausible impacts if a scenario of price increase in high sugar snack were to be implemented and suggests that this option is worthy of further research and consideration as part of an integrated approach to tackling obesity.

**What is already known on this topic**

Taxation strategies to lower sugar and energy intake have focused on sugar sweetened beverages; in the UK, high sugar snacks, such as confectionery, make a more substantial contribution to intakes of free sugars and energy than do sugar sweetened beverages
Encouraged by the large reformulation efforts of the food industry after the Soft Drink Industry Levy was introduced, Public Health England developed a voluntary sugar reduction and reformulation programme for snacks, showing modest results after the first year and highlighting the need for additional interventions to reduce sugar intake through high sugar snacks
Several countries, including Mexico, Finland, and Hungary introduced taxes on unhealthy foods, including high sugar snacks: early evaluations show a major reduction in the purchase of such foods

**What this study adds**

Our study suggests that a 20% price increase in high sugar snacks has the potential to reduce overall energy purchased among all body mass index and income groups in the UK, leading to an estimated population level reduction in obesity prevalence of 2.7 percentage points after the first year
The results of this study also suggest that price increases in high sugar snacks could also make an important contribution to reducing health inequalities driven by diet related disease
Our analysis provides policymakers with estimates of the relative magnitude of plausible impacts of a scenario of price increase in high sugar snacks and suggests that this option is worthy of further research and consideration as part of an integrated approach to tackling obesity

## Web extra

Extra material supplied by authors

**Supplementary information:** appendices 1-6

**Supplementary information:** technical appendix

## Notes

Contributors: PFDS did the literature search, analysed and interpreted the data, and wrote the manuscript. LC did the literature search, designed the study, analysed and interpreted the data, and commented on the manuscript. PFDS and LC are guarantors. TMM designed the study, interpreted the data, and commented on the manuscript. SAJ designed the study, interpreted the data, and commented on the manuscript. RS designed the study, analysed and interpreted the data, and commented on the manuscript. The final version of the report and ultimate decision to submit for publication was determined by the authors. The corresponding author attests that all listed authors meet authorship criteria and that no others meeting the criteria have been omitted.

Ethical approval: Not required as the data used in this study are secondary, anonymised purchase and open access data. No patients were involved in the study.

Data sharing: No additional data available.

The lead author (PFDS) affirms that this manuscript is an honest, accurate, and transparent account of the study being reported; that no important aspects of the study have been omitted; and that any discrepancies from the study as planned (and, if relevant, registered) have been explained.
