# Supplementary material for: Potential impact on prevalence of obesity in the UK of a 20% price increase in high sugar snacks: modelling study
Source: BMJ. 2019 Sep 4;366:l4786. doi: 10.1136/bmj.l4786 (PMC6724407; doi:10.1136/bmj.l4786)
Supplement: Supplementary file 1 — Supplementary information: appendices 1-6 [file schp048338.ww1.pdf]

Appendix 1

A. Descriptive characteristics of sample population

Characteristics	Household BMI status								
	Non-overweight			Overweight			Obese		
	Low *	Middle	High	Low	Middle	High	Low	Middle	High
Number of observations	69,775	107,647	39,001	67,232	91,507	26,226	60,197	64,522	15,744
Number of households	3,543	5,455	1,984	3,271	4,502	1,309	2,997	3,260	794
Age of main purchaser (SE)	51.2 (17.9)	44.3 (14.5)	41.3 (10.6)	56.2 (16.6)	48.7 (14.5)	44.5 (11.0)	53.9 (15.4)	48.6 (13.9)	45.2 (10.3)
Household size (SE)	2.2 (1.3)	2.9 (1.3)	3.15(1.2)	2.2 (1.2)	2.8 (1.3)	3.2 (1.2)	2.2 (1.2)	2.8 (1.3)	3.1 (1.2)
Share of households that have children (%)	30.3	45.5	51.2	24.0	38.4	48.7	24.4	36.3	42.9
Number of children if>0 (SE)	1.7 (0.8)	1.8 (0.9)	1.7 (0.7)	1.8 (0.9)	1.8 (0.8)	1.7 (0.8)	1.7 (0.9)	1.8 (0.9)	1.7 (0.8)
**Social Class (%)**									
Class A and B (highly skilled)	6.2	23.1	54.1	6.9	21.8	52.5	4.6	18.3	50.1
Class C1	34.9	43.6	36.1	31.6	42.1	36.8	25.2	41.8	34.3
Class C2	15.7	21.6	8.3	16.1	22.9	8.7	15.2	23.3	13.2
Class D	21.6	10.4	1.2	21.8	11.4	1.8	21.4	13.7	2
Class E (unemployed, retired)	21.7	1.4	0.3	23.6	1.8	0.2	33.6	2.8	0.4
**Highest Qualification (%)**									
Degree or higher	13.6	30	51	12.7	25.6	48.2	9.8	22.5	44.8
Higher education	12.3	15.9	12	11.7	15.9	12.8	12	16.4	12.2
A level	11.6	14.1	11	10.1	13.8	10.8	8.5	12.7	10.8
Secondary education (GCSE)	22.8	17.5	9.9	21.9	20.7	11.8	24.02	21.3	14.7
Other	11.2	5	2.8	13.3	7.3	3.6	12.9	7.7	3.5
None	13.4	3.2	0.7	17.9	4.6	1.5	19	6.1	1
Unknown	15.2	14.3	12.8	12.5	12.2	11.3	13.8	13.3	12.9
**Tenure (%)**									
Owned outright	32.6	23	17.4	36.9	27.7	17	26.4	23.7	16.9
Mortgaged	20.5	51.8	71.9	16.8	48.2	71.5	15	45.7	71
Rented	44.6	23.5	9.4	44	22.5	10.8	56.6	28.9	10.6
Other	2.1	1.5	0.9	2	1.4	0.5	1.7	1.2	1
Unknown	0.3	0.2	0.3	0.3	0.2	0.2	0.4	0.5	0.5
**Body Mass Index (SD)**	22.1 (2.1)	22.2 (2.0)	22.2 (1.9)	27.3 (1.4)	27.2 (1.4)	27.2 (1.4)	35.3 (5.1)	34.8 (4.7)	34.2 (4.0)

*** Low (<£20,000) - Middle (£20,000 – 49,999) - High (≥£50,000)**

B. own- price elasticities per income and BMI group

Food group	Household BMI status & Income group								
	**Non-overweight**			**Overweight**			**Obese**		
	Low	Middle	High	Low	Middle	High	Low	Middle	High
High sugar drinks	-0.819	-0.880	-0.564	-0.843	-0.623	-0.674	-0.815	-0.753	-0.307
Medium sugar drinks	-0.609	-0.775	-0.674	-0.465	-0.615	-0.416	-0.661	-0.610	-0.823
Low sugar drinks	-1.057	-0.781	-0.643	-0.580	-0.852	-0.658	-0.877	-0.804	-0.769
Other drinks	-0.803	-0.729	-0.761	-0.944	-0.760	-0.922	-0.926	-1.101	-0.783
Alcohol	-0.982	-0.951	-0.776	-0.861	-0.949	-0.831	-0.885	-0.795	-0.925
Biscuits	-0.634	-0.578	-0.799	-0.852	-0.731	-0.695	-0.705	-0.825	-0.341
Confectionary	-0.630	-0.824	-0.688	-0.792	-0.665	-0.697	-0.735	-0.717	-0.775
Cakes	-0.745	-0.536	-0.632	-0.524	-0.612	-0.294	-0.914	-1.042	-0.712
Savoury snacks	-0.682	-0.743	-0.873	-0.604	-0.781	-0.685	-0.741	-0.673	-0.871
Meat	-0.781	-0.842	-0.732	-0.733	-0.693	-0.757	-0.838	-0.691	-0.447
Dairy	-0.881	-0.871	-1.052	-0.906	-0.887	-0.880	-0.805	-0.923	-0.752
Fruit and vegetables	-0.605	-0.573	-0.693	-0.561	-0.595	-0.699	-0.618	-0.574	-0.568
Other (aggregate)	-0.499	-0.680	-0.643	-0.501	-0.641	-0.641	-0.450	-0.697	-0.712

C. Baseline energy (kcal) purchases per household member per day*

Food group	Household BMI status and income group																	
	Non-overweight						Overweight						Obese					
	Low		Mid		High		Low		Mid		High		Low		Mid		High	
	mean	SE	mean	SE	mean	SE	mean	SE	mean	SE	mean	SE	mean	SE	mean	SE	mean	SE
High sugar drinks	25.9	1.1	23.9	0.7	19.3	0.9	25.8	1.0	24.0	0.7	22.9	1.3	28.3	1.2	25.9	0.9	25.2	1.7
Medium sugar drinks	2.1	0.1	1.9	0.1	1.8	0.1	2.6	0.1	2.3	0.1	2.1	0.2	2.6	0.1	2.4	0.1	2.5	0.3
Low sugar drinks	5.3	0.2	5.8	0.1	5.5	0.2	5.9	0.2	6.0	0.1	6.2	0.3	6.9	0.2	6.6	0.2	7.5	0.4
Other drinks	15.9	0.5	18.3	0.4	22.2	0.8	15.6	0.6	17.8	0.5	22.2	1.0	15.5	0.6	15.2	0.5	20.1	1.2
Alcohol	77.9	3.5	73.2	2.1	66.3	2.9	84.1	3.5	80.1	2.4	74.4	3.7	67.7	3.2	75.8	3.2	82.6	5.3
Biscuits	109.9	2.6	91.8	1.6	72.9	2.2	119.0	2.7	94.6	1.7	77.9	2.9	122.4	2.8	96.4	2.1	86.3	4.0
Confectionary	72.4	1.8	57.4	1.1	52.0	1.6	84.2	2.1	62.2	1.3	55.5	2.3	87.9	2.1	70.7	1.9	68.4	3.5
Cakes	28.5	0.9	23.7	0.6	19.1	0.8	31.2	1.0	24.2	0.6	22.0	1.2	31.2	1.0	24.0	0.7	23.5	1.4
Savoury snacks	64.4	1.4	67.1	1.2	63.7	1.7	72.5	1.7	69.5	1.2	66.7	2.1	79.2	1.8	72.0	1.5	78.1	3.4
Meat	160.5	3.7	142.3	2.2	144.2	3.6	179.4	3.7	161.5	2.5	165.0	5.1	180.9	3.8	167.2	3.2	172.0	6.1
Dairy	394.1	6.9	327.1	4.3	315.3	7.0	430.2	7.6	333.4	4.4	307.8	7.8	419.7	7.2	338.7	5.6	336.6	10.6
Fruit and vegetables	165.1	3.5	143.9	2.1	149.1	3.4	179.3	3.5	145.7	2.1	149.7	4.7	162.3	3.3	142.4	2.7	143.8	4.9
Other (aggregate)	1092.6	16.6	924.9	10.8	877.6	17.1	1176.5	18.2	959.3	11.7	908.8	22.2	1175.7	18.8	967.7	14.4	971.1	30.4
Total	2214.6	32.4	1901.3	21.4	1808.7	34.3	2406.3	36.0	1980.6	32.2	1881.2	44.4	2380.2	36.4	2004.8	29.1	2017.6	60.0

**estimated from 2013 data as weighted daily average. Weights were provided with the data and account for both under-reporting (response weight) across shop types and food products as well as sampling (demographic weight). Daily average was calculated by first estimating response-weighted household annual totals across the food groups, which were then divided by household size and multiplied by normalized average demographic weight (ranging from 0 to 1).*

D. Notes to own price-elasticities per income group.

Full basket - *The analysis consists of a full food basket of foods and beverages purchased and taken home. All foods other than those consumed out-of-home are included in the database. Anything that is not included in a named category is classified under "other".*

Meat elasticities - *While meat is a frequently consumed food in British diets, it is relatively more expensive compared to many other foods. This could explain why the demand is relatively more responsive to price changes. Furthermore, it compares well to estimates from other sources. The most recent data reported by Tiffin et al (2011- (1)) show a* ***meat elasticity of -0.804****, comprehensively covering full food baskets estimated from Living Cost and Food Survey data.*

Fruit and vegetables & dairy elasticities - *Also other elasticities show similarities with previous studies: elasticity for fruits and vegetables of -0.698 and -0.633, respectively, which is very similar to our estimates. Our estimates are slightly bigger for dairy elasticities (around -0.8 to -0.9 in comparison to -0.51 reported by Tiffin); which might be partly explained by the fact that we did not combine the eggs and dairy categories.*

Living Cost and Food Survey *- Briggs et al 2013 (2) utilise elasticities estimated from Living Cost and Food Survey Data (3) for their estimates on sugary drinks. Their findings are comparable to the findings of this study. They found a diet soft drink own price elasticity of -0.903 compared to our overall estimate of -0.780. For sugary beverages they report -0.811 and our estimate is -0.697. For juice and water they report own elasticity between -0.971 to -1.174 and our estimate is -0.859; For alcohol, Briggs and colleagues report elasticities between -0.921 to -1.01 whereas we find -0.884.*

Conclusions - *While some difference exist, the relative responsiveness of foods and drinks is similar: sugary drinks are least responsive, followed by diet drinks and then other beverages. It should be noted that estimated elasticities are using data from similar period (Briggs and colleagues use 2010 data), but that despite different data sources and modelling approaches estimates correspond relatively well. We added a few lines on this observation as well.*

References

1. *Tiffin R, Balcombe K, Salois M, Kehlbacher AJUoR. Estimating food and drink elasticities. 2011.*
2. *Briggs AD, Mytton OT, Kehlbacher A, Tiffin R, Rayner M, Scarborough PJB. Overall and income specific effect on prevalence of overweight and obesity of 20% sugar sweetened drink tax in UK: econometric and comparative risk assessment modelling study. 2013;347:f6189.*
3. *Department for the Environment Food and Rural Affairs. Living Costs and Food Survey 2010. 2011.*

Appendix 2

Sensitivity analysis – Households without children

A. Changes in energy purchase by price increase scenario: households without children versus all households - stratified by income group

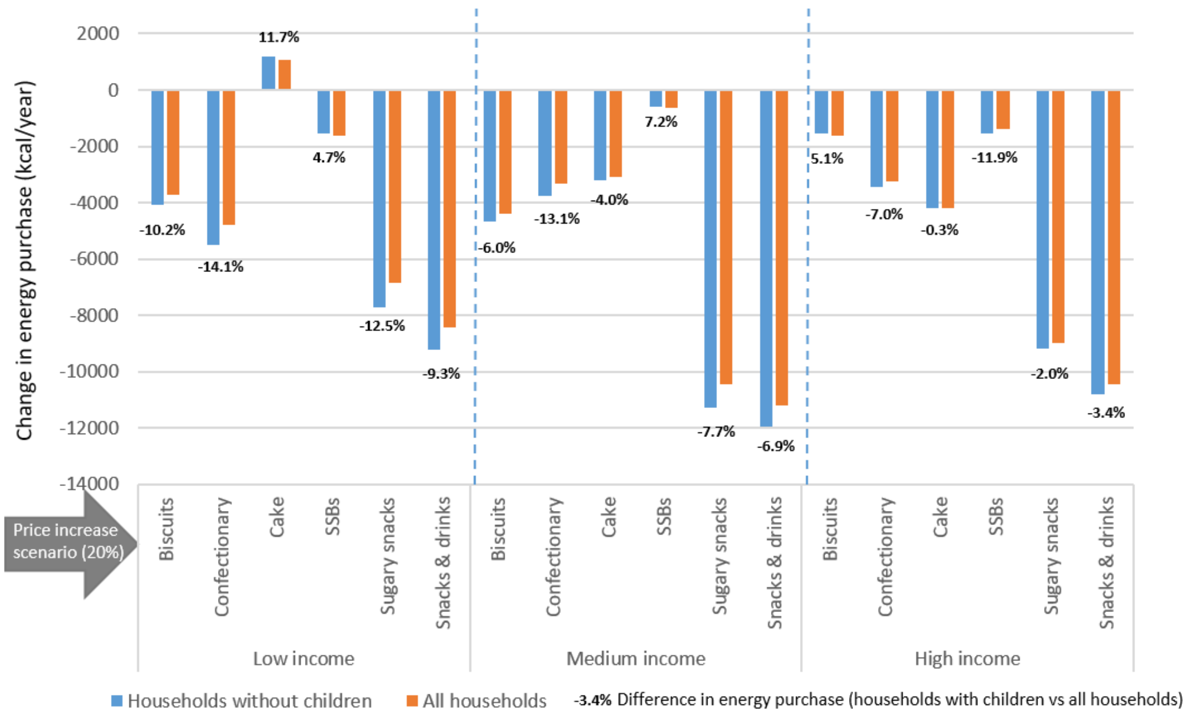

B. Changes in energy purchase by price increase scenario: households without children versus all households - stratified by income and BMI group

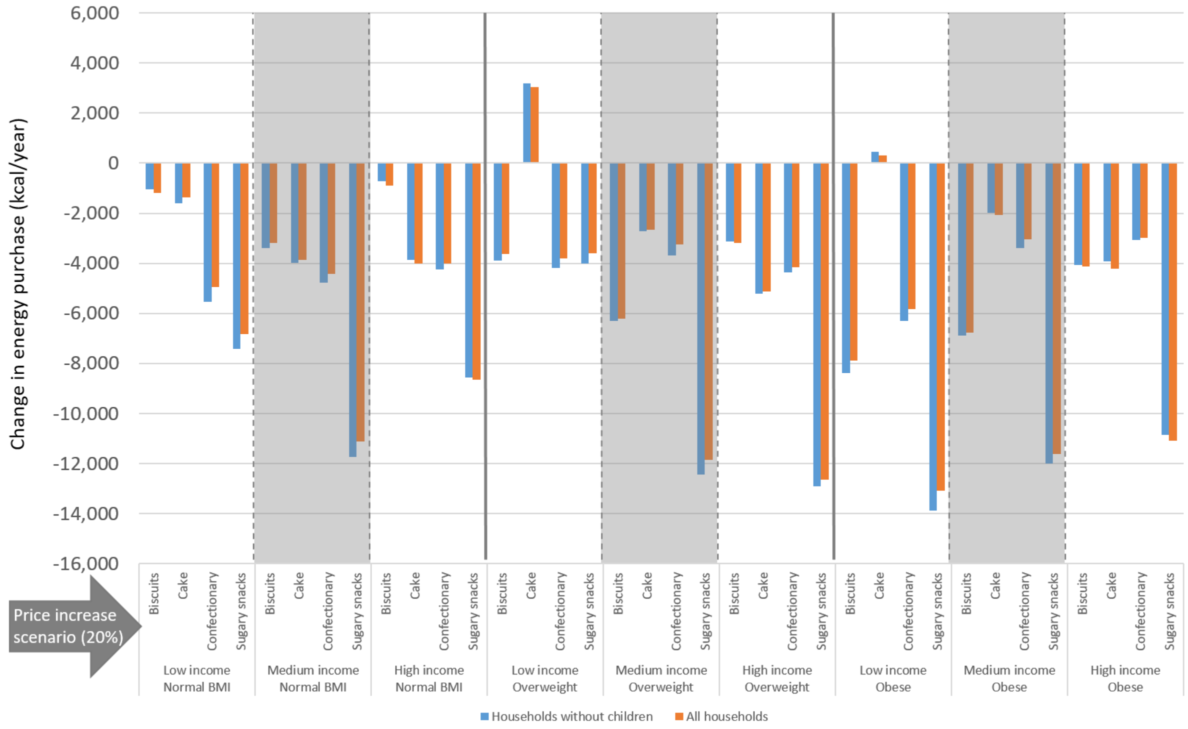

Appendix 3

Analysis with Bonferroni correction

Change in energy purchase by price increase scenario: analysis stratified by BMI and income group

BMI category	Income group	Change in energy purchase (kcal [95% CI]) by price increase scenario			
		20% price increase in biscuits	*20% price increase in cakes*	*20% price increase in confectionary*	*20% price increase in all high-sugar snacks*
Not overweight	*Low income*	-1188.96 *[-1677 to -701]*	-1378.33 *[-1786 to -971]*	-4956.9 *[-5630 to -4284]*	-6819.68 *[-8360 to -5279]*
	Middle income	-3197.52 *[-3748 to -2647]*	-3876.3 *[-4795 to -2957]*	-4434.21 *[-4934 to -3935]*	-11126.9 *[-12997 to -9257]*
	High income	-893.09 *[-1415 to -371]*	-4014.04 *[-5830 to -2199]*	-4006.25 *[-4804 to -3208]*	-8635.76 *[-11427 to -5844]*
Overweight	*Low income*	-3634.78 *[-4598 to -2671]*	3023.113 *[2251 to 3795]*	-3797.6 *[-4410 to -3185]*	-3601.24 *[-4750 to -2452]*
	Middle income	-6210.92 *[-7213 to -5209]*	-2659.19 *[-3321 to -1997]*	-3252.04 *[-3635 to -2869]*	-11841.4 *[-13828 to -9855]*
	High income	-3188.9 *[-4245 to -2133]*	-5133.04 *[-7570 to -2696]*	-4144.5 *[-5091 to -3198]*	-12628.4 *[-16689 to -8568]*
Obese	*Low income*	-7877.83 *[-9461 to -6294]*	313.1469 *[80 to 546]*	-5833.26 *[-6642 to -5025]*	-13075.9 *[-15650 to -10501]*
	Middle income	-6763.04 *[-7926 to -5600]*	-2065.07 *[-2641 to -1489]*	-3051.85 *[-3507 to -2596]*	-11615.3 *[-13733 to -9498]*
	High income	-4120.17 *[-5493 to -2748]*	-4206.1 *[-6364 to -2048]*	-2981.67 *[-3846 to -2117]*	-11080.2 *[-15070 to -7090]*

Appendix 4

Changes in BMI among overweight and obese households

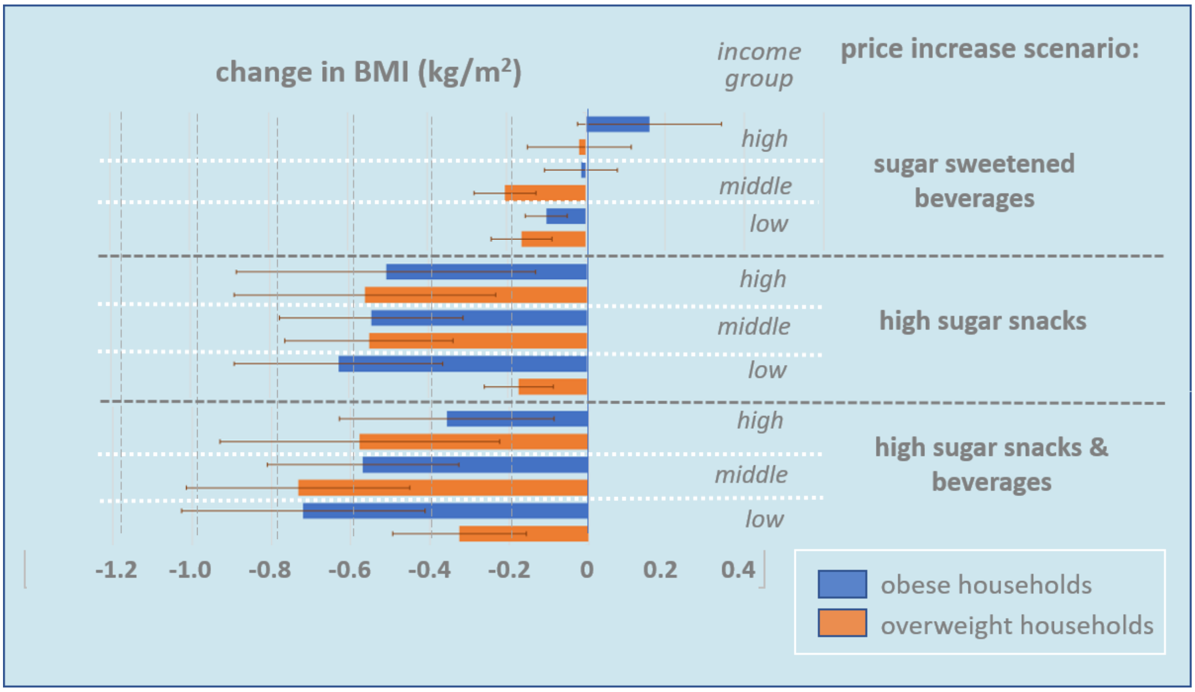

Changes in BMI (kg/m2) by income and pre-existing BMI 12 months after the introduction of a 20% price increase

A combined price increase of high-sugar snacks and drinks would decrease BMI on average by -0.53 kg/m2 [95% CI: -1.01 to -0.06] pooling together all BMI and income groups. In the households classified as obese this ranged from -0.36kg/m2 [95% CI: -0.63 to -0.09] for households in the high-income group to -0.72 kg/m2 [95% CI: -1.03 to -0.41] for households in the high-income group – a year after the introduction of such price increase.

Appendix 5

Changes in prevalence of non-overweight and obesity

		Non-overweight		Obesity	
**Price increase scenario**	Income level	Change in Prevalence (% points)	P-value*	Change in Prevalence (% points)	P-value*
**Sugar sweetened beverages**	low	+1.43	*	-0.22	
	middle	+1.41		-0.08	
	high	-0.94		0.00	
**All high sugar snacks**	low	+1.65	*	-3.07	**
	middle	+4.15	**	-2.49	**
	high	+3.73	**	-2.34	*
**High sugar beverages & snacks**	low	+2.53	*	-3.62	**
	middle	+5.73	**	-2.74	**
	high	+4.20	**	-1.17	*
**Based on 1000 MC simulations, considering variance in weight change and population variability: *=<0.05 **=<0.01*					

Appendix 6

Market size per food group. Source: ©Euromonitor International

Market size per capita (2014)	UK	USA	Mexico	Australia
**Confectionery, sweet biscuits, snack bars, fruit snacks (kg)**	19.3	14.3	7.3	15.5
**Soft drinks (including carbonates, bottled water, concentrates, juices, energy drinks) (l)**	161.3	346	225	170.6
**Market size snacks ratio (as compared to UK)**	-	**0.74**	**0.38**	**0.80**
**Market size drinks ratio (as compared to UK)**	-	**2.14**	**1.40**	**1.06**
**Drinks to snack ratio**	8.4	24.2	30.8	11.0
**Drinks to snack ratio of ratios as compared to UK**	-	2.88	3.67	1.31
