# Supplementary material for: Potential impact on prevalence of obesity in the UK of a 20% price increase in high sugar snacks: modelling study
Source: BMJ. 2019 Sep 4;366:l4786. doi: 10.1136/bmj.l4786 (PMC6724407; doi:10.1136/bmj.l4786)

Technical appendix

1. Demand modelling strategy

The demand model applied follows closely that used by Smith et al (2018). It is based on the linear version of Almost Ideal Demand System where expenditure shares are modelled as a function of prices and total expenditure (as an approximation for income) adjusted for all price levels (1):

$$w_{iht} = \alpha_i + \sum_{j=1}^{N} \gamma_{ij} lnp_{jht} + \beta_i \frac{lnx_{ht}}{P_{ht}} + \varepsilon_{iht} \quad \text{[Equation 1]}$$

where:

w_{iht} is expenditure share of group *i* (i=1, 2, …, 13) for household *h* (h=1,2,…27,115) in 4-weekly periods *t* (t=1, 2, …, 26)

lnx_{ht} is the log of total household monthly expenditure on food and beverage per capita

lnp_{jht} is the log of price for category *j* for household *h* in period *t*

P_{ht} is a Laspeyres price index of geometrically weighted average prices defined as $lnP = \sum_i \overline{w}_i lnp_i$

ε_{iht} is a random disturbance

To deal with zero observations that can bias the estimates, we followed a two-step procedure developed by Deaton (2). In the first step, the decision to purchase beverages in any group was modelled as a function of lagged quantity (L) of beverages purchased in that group, household size, age of the main shopper, socio-economic group (A&B, C1&C2 or D&E), whether or not the household owns their house, income group (for the whole sample only), presence of children and time indicators to take into account seasonal trends, using a probit model. From the probit model, we estimated the probability density function (ϕ_i) and cumulative density function (Φ_i) of the predictions of the fitted model. These two variables were applied in the second step of estimating the demand function (2):

$$w^*_{iht} = \Phi_{iht}(w_{iht}) + \varphi_i \phi_{iht} + \sum_{t=1}^{13} \rho_{it} T_{it} + v_{ih} + \varepsilon_{it} \quad \text{[Equation 2]}$$

T_{it} are indicator variables to capture any seasonal or other time effects (13 four-week periods)

v_{ih} is a fixed household effect

For each of the thirteen groups i=1, 2 ,…,13 we estimated equation-by-equation using a fixed effect model with robust clustered standard errors to allow for any misspecification, particularly serial correlation of observations within the households (equation [2]. Clusters were defined at the geographical area used in estimating prices (n=110).

The specification used [equation 2] imposed the restrictions, compatible with the AIDS model, of adding-up $[\sum_{i=1}^{N} \alpha_i = 1;\ \sum_{i=1}^{N} \beta_i = 0]$ and homogeneity $[\sum_{i=1}^{N} \gamma_{ij} = 0]$.

There are two important sources of potential endogeneity in the model. First, total expenditure enters the model as a proxy for incomes, while it is also used to calculate the expenditure shares. Furthermore, total expenditure might be endogenous because of possible correlation with unobserved characteristics affecting demand behaviour or because of shocks common to total expenditure and expenditure shares. Secondly, unit prices estimated from monthly aggregates of expenditure and volume are likely to be biased due to aggregation effects (2). If prices or expenditures are correlated with the equation errors, estimators will be both biased and inconsistent.

To deal with quality effects in prices, we took the assumption that in a relatively small geographical area households face the same prices during the same time period (3). To estimate these geographical average unit values we calculated the monthly average prices for the (n=110) postcode areas which

we observe in the data. Where the monthly price was missing (e.g. households did not purchase the products in this beverage group in a particular month), it was replaced by the first non-missing average of the previous and the following monthly prices.

To reduce possible endogeneity between expenditure shares (w_{iht}) and total expenditure (lnx_{ht}) that enters the demand equation in equation 1, we use the approach developed in by Smith *et al* (4) and regressed household per capita expenditure (lnx_{ht}) on household socio demographic characteristics (social class, income, income squared (whole sample only), household size and presence of children. The predicted values from the model were used as instruments for total expenditure (lnx_{ht}) in equation 1.

The demand model was estimated in nine sub-samples based on three household income categories (<£20,000; £20,000 - £49,000, >=£50,000 per year) and three BMI categories (<25; ≥25 & <30; ≥30).

Uncompensated elasticities were estimated for beverages and individual beverage groups, at sample averages from model estimates of each of the sub-samples as follows:

$$e_{ij} = \Phi_i * \left(\frac{\gamma_{ij}}{w_i} - \frac{\beta_i w_j}{w_i}\right) - \Delta_{ij} \qquad \text{[Equation 3]}$$

Where Δ_{ij} is the Kronecker delta which equals 1 when *i=j* and 0 otherwise.

References

1. Shonkwiler JS, Yen STJAJoAE. Two-step estimation of a censored system of equations. 1999;81(4):972-82.

2. Deaton A. Quality, quantity, and spatial variation of price. 1988:418-30.

3. Blundell R, Robin J. Estimation in large and disaggregated demand systems: An estimator for conditionally linear systems. 1999;14(3):209-32.

4. Smith RD, Cornelsen L, Quirmbach D, Jebb SA, Marteau TM. Are sweet snacks more sensitive to price increases than sugar-sweetened beverages: analysis of British food purchase data. BMJ open. 2018;8(4):e019788.

2. Price elasticities of demand

		Not overweight			Overweight			Obese		
Demand changes	price changes	Low-inc	Mid-inc	High-inc	Low-inc	Mid-inc	High-inc	Low-inc	Mid-inc	High-inc
sugary drink	sugary drink	**-0.819**	**-0.880**	**-0.564**	**-0.843**	**-0.623**	**-0.674**	**-0.815**	**-0.753**	**-0.307**
med-sug drink	sugary drink	-0.377	-0.769	-0.450	-0.013	0.287	-0.466	-0.012	-0.016	-0.269
low-sug drink	sugary drink	-0.025	-0.011	-0.016	-0.156	0.021	-0.101	0.161	-0.028	-0.055
other drink	sugary drink	0.105	0.081	0.013	0.072	0.103	0.317	-0.090	0.191	0.081
alcohol	sugary drink	-0.035	-0.042	-0.053	0.037	0.023	-0.195	-0.009	-0.177	-0.159
biscuits	sugary drink	0.020	-0.035	-0.161	-0.050	-0.096	0.132	0.013	0.196	0.001
confec	sugary drink	0.055	0.129	0.233	0.224	-0.008	-0.001	-0.021	-0.051	0.064
cakes	sugary drink	-0.170	0.127	-0.451	-0.245	-0.067	-0.180	0.043	0.295	0.493
sav snacks	sugary drink	0.018	0.001	0.167	-0.038	-0.030	-0.192	0.082	-0.055	0.090
meat	sugary drink	-0.030	-0.012	-0.033	0.047	-0.064	0.046	0.009	0.061	-0.034
dairy	sugary drink	-0.031	0.028	0.059	0.012	0.022	-0.011	0.067	0.077	-0.016
fruit and veg	sugary drink	-0.002	0.027	0.017	-0.046	-0.011	0.039	-0.064	-0.007	-0.133
other	sugary drink	0.029	0.001	-0.017	-0.032	-0.022	0.042	-0.034	-0.022	0.052
sugary drink	med-sug drink	-0.067	-0.066	0.038	-0.050	0.024	-0.001	-0.001	-0.047	-0.139
med-sug drink	med-sug drink	**-0.609**	**-0.775**	**-0.674**	**-0.465**	**-0.615**	**-0.416**	**-0.661**	**-0.610**	**-0.823**
low-sug drink	med-sug drink	0.038	-0.043	-0.060	-0.034	-0.063	0.032	-0.017	-0.040	0.035
other drink	med-sug drink	-0.011	-0.080	-0.082	0.081	-0.026	-0.126	-0.076	-0.055	0.069
alcohol	med-sug drink	0.011	-0.011	-0.072	-0.068	-0.018	0.036	-0.063	-0.011	-0.102
biscuits	med-sug drink	0.007	-0.008	-0.036	-0.025	0.008	0.072	-0.037	0.031	0.046
confec	med-sug drink	0.008	-0.027	0.065	0.008	0.056	0.060	0.013	0.035	0.009
cakes	med-sug drink	-0.033	0.060	-0.101	-0.064	0.007	0.033	-0.077	0.027	-0.125
sav snacks	med-sug drink	0.036	-0.005	-0.031	0.020	0.015	0.027	0.001	0.050	0.020
meat	med-sug drink	-0.016	0.007	-0.009	-0.002	0.002	-0.009	0.027	0.007	0.011
dairy	med-sug drink	-0.004	-0.004	-0.002	-0.008	-0.020	-0.031	-0.013	-0.017	0.053
fruit and veg	med-sug drink	0.005	0.014	-0.017	0.007	0.018	-0.027	-0.010	-0.004	-0.015
other	med-sug drink	-0.016	0.006	0.041	0.012	-0.009	-0.019	0.021	-0.007	0.016
sugary drink	low-sug drink	-0.109	-0.140	-0.381	-0.318	0.071	-0.300	-0.014	-0.065	-0.301
med-sug drink	low-sug drink	-0.441	-0.586	-0.408	-0.161	-0.566	0.195	-0.153	-0.342	0.889
low-sug drink	low-sug drink	**-1.057**	**-0.781**	**-0.643**	**-0.580**	**-0.852**	**-0.658**	**-0.877**	**-0.804**	**-0.769**
other drink	low-sug drink	0.189	-0.015	0.021	0.018	0.106	-0.064	0.124	-0.188	-0.440
alcohol	low-sug drink	-0.027	0.145	-0.143	0.044	-0.006	0.019	-0.064	-0.140	0.181
biscuits	low-sug drink	0.092	0.114	0.019	0.157	0.229	0.370	-0.002	0.306	-0.103
confec	low-sug drink	0.143	0.227	0.016	0.086	0.262	-0.054	0.193	0.221	-0.052
cakes	low-sug drink	0.342	0.095	0.160	0.299	0.048	-0.173	0.189	0.297	0.266
sav snacks	low-sug drink	-0.125	0.102	-0.128	0.001	-0.074	0.027	0.019	0.053	0.037
meat	low-sug drink	-0.023	-0.022	0.022	0.028	0.045	-0.038	-0.026	0.079	-0.026
dairy	low-sug drink	0.014	0.043	0.013	-0.060	0.041	-0.020	0.013	0.009	-0.033
fruit and veg	low-sug drink	0.067	-0.071	0.072	0.089	0.019	-0.054	-0.068	-0.003	-0.026
other	low-sug drink	-0.018	-0.069	0.025	-0.078	-0.079	0.002	0.005	-0.031	-0.042
sugary drink	other drink	-0.014	-0.013	-0.047	0.011	-0.119	-0.142	-0.022	-0.094	0.203
med-sug drink	other drink	-0.522	-0.280	-0.436	0.208	-0.536	-0.178	-0.572	-0.645	0.003
low-sug drink	other drink	-0.174	-0.063	-0.224	-0.156	-0.060	-0.379	-0.096	-0.045	-0.062
other drink	other drink	**-0.803**	**-0.729**	**-0.761**	**-0.944**	**-0.760**	**-0.922**	**-0.926**	**-1.101**	**-0.783**
alcohol	other drink	-0.092	-0.139	-0.194	0.016	-0.142	-0.169	-0.042	-0.007	0.188
biscuits	other drink	0.002	0.018	0.127	0.022	0.070	0.089	0.168	0.042	0.011
confec	other drink	-0.060	0.052	-0.011	0.058	0.063	-0.039	0.106	-0.011	0.073
cakes	other drink	0.106	0.155	0.213	0.226	-0.041	-0.257	0.100	0.033	-0.130
sav snacks	other drink	-0.012	-0.112	-0.060	0.068	-0.063	-0.069	-0.007	0.003	0.025
meat	other drink	0.057	0.008	0.061	-0.007	0.041	0.041	0.031	-0.015	-0.192
dairy	other drink	-0.003	0.005	0.031	-0.003	0.077	0.048	0.102	0.025	0.101
fruit and veg	other drink	-0.035	-0.037	0.042	-0.003	-0.031	0.099	-0.018	-0.017	-0.042
other	other drink	0.056	0.058	0.030	-0.027	0.040	0.057	-0.025	0.043	-0.048
sugary	alcohol	-0.206	-0.098	-0.134	-0.174	-0.231	-0.204	-0.385	-0.164	-0.283
med-sugar	alcohol	-0.464	-0.001	-0.046	-0.219	0.088	-0.083	-0.076	-0.486	-0.866
low-sugar	alcohol	-0.233	-0.272	-0.247	-0.330	-0.200	-0.155	-0.245	-0.253	-0.246

other drink	alcohol	-0.291	-0.192	-0.106	-0.363	-0.149	-0.109	-0.274	-0.076	0.113
alcohol	alcohol	**-0.982**	**-0.951**	**-0.776**	**-0.861**	**-0.949**	**-0.831**	**-0.885**	**-0.795**	**-0.925**
biscuits	alcohol	0.040	0.115	0.029	-0.053	0.136	0.063	-0.025	0.074	0.079
confec	alcohol	-0.008	0.037	0.023	0.099	0.058	0.214	0.099	0.065	0.260
cakes	alcohol	-0.075	-0.031	-0.031	-0.102	0.034	0.188	-0.108	0.079	0.246
sav snacks	alcohol	-0.068	0.007	0.016	-0.012	-0.078	-0.028	-0.026	-0.078	-0.155
meat	alcohol	0.072	0.064	-0.001	0.005	0.043	-0.034	0.074	0.024	-0.004
dairy	alcohol	0.022	0.007	-0.043	0.012	-0.001	0.009	0.024	-0.010	-0.044
fruit and veg	alcohol	-0.049	-0.001	-0.009	-0.018	-0.011	-0.058	-0.018	0.020	-0.016
other	alcohol	0.060	-0.014	-0.032	0.013	-0.003	-0.026	0.003	-0.040	0.035
sugary drink	biscuits	-0.023	0.050	-0.047	-0.094	0.150	0.169	0.131	-0.145	-0.116
med-sug drink	biscuits	-0.399	-0.289	-0.600	0.142	0.054	-0.621	-0.097	0.355	-0.584
low-sug drink	biscuits	-0.028	-0.035	-0.123	-0.011	-0.029	-0.044	0.065	0.040	0.214
other drink	biscuits	-0.058	0.091	0.049	0.096	0.190	0.081	-0.119	-0.275	-0.288
alcohol	biscuits	-0.077	-0.108	-0.150	-0.088	-0.002	-0.167	0.070	0.015	0.015
biscuits	biscuits	**-0.634**	**-0.578**	**-0.799**	**-0.852**	**-0.731**	**-0.695**	**-0.705**	**-0.825**	**-0.341**
confec	biscuits	-0.368	-0.074	-0.430	-0.488	-0.018	0.198	0.048	-0.132	-0.353
cakes	biscuits	-0.316	-0.306	-0.289	-0.155	-0.221	-0.594	0.039	-0.125	-0.024
sav snacks	biscuits	-0.092	-0.013	-0.078	-0.138	-0.102	0.015	0.086	-0.003	-0.035
meat	biscuits	-0.064	-0.009	0.048	-0.033	-0.131	-0.012	-0.163	0.026	-0.065
dairy	biscuits	0.058	0.006	0.057	0.012	0.045	-0.067	-0.026	0.022	0.030
fruit and veg	biscuits	0.046	0.013	0.082	0.025	0.057	0.155	-0.045	0.001	0.077
other	biscuits	0.076	0.027	0.061	0.095	-0.012	0.021	0.006	-0.007	-0.002
sugary drink	confec	-0.034	-0.069	-0.106	-0.137	-0.118	-0.061	0.024	0.007	-0.125
med-sug drink	confec	-0.100	0.007	0.075	-0.101	-0.106	0.096	-0.215	-0.225	-0.037
low-sug drink	confec	-0.097	-0.112	0.019	-0.036	-0.028	-0.112	-0.072	-0.074	-0.024
other drink	confec	-0.017	-0.035	-0.112	-0.074	-0.040	-0.123	-0.025	-0.098	-0.169
alcohol	confec	0.017	0.069	0.044	0.007	-0.006	0.094	0.021	-0.002	-0.024
biscuits	confec	-0.136	-0.116	-0.088	-0.081	-0.062	-0.213	-0.138	-0.151	-0.047
confec	confec	**-0.630**	**-0.824**	**-0.688**	**-0.792**	**-0.665**	**-0.697**	**-0.735**	**-0.717**	**-0.775**
cakes	confec	-0.095	-0.239	-0.203	-0.059	-0.145	-0.049	-0.243	-0.112	-0.243
sav snacks	confec	-0.057	0.003	-0.096	-0.029	0.027	-0.098	0.038	-0.047	-0.022
meat	confec	0.031	0.003	0.014	-0.004	0.001	-0.027	-0.004	-0.041	-0.022
dairy	confec	0.004	-0.011	-0.029	-0.030	-0.031	-0.027	-0.034	0.004	-0.059
fruit and veg	confec	-0.093	-0.053	-0.056	-0.105	-0.072	-0.077	-0.059	-0.063	-0.016
other	confec	0.008	0.012	0.016	0.056	0.031	0.031	0.026	0.048	0.060
sugary drink	cakes	0.149	-0.054	0.156	-0.231	0.006	0.057	-0.030	0.007	0.002
med-sug drink	cakes	0.278	-0.003	-0.329	-0.867	-0.331	-0.413	0.111	0.250	-0.381
low-sug drink	cakes	-0.048	0.013	-0.006	0.018	0.070	0.097	-0.127	-0.139	-0.048
other drink	cakes	0.034	0.058	0.170	0.027	-0.115	-0.030	-0.096	-0.060	-0.048
alcohol	cakes	-0.030	0.128	0.057	-0.050	0.153	0.222	0.003	0.128	0.218
biscuits	cakes	-0.022	-0.125	-0.042	0.029	-0.134	-0.053	-0.059	0.015	-0.147
confec	cakes	-0.116	-0.012	0.130	0.087	0.033	0.485	0.020	0.113	0.128
cakes	cakes	**-0.745**	**-0.536**	**-0.632**	**-0.524**	**-0.612**	**-0.294**	**-0.914**	**-1.042**	**-0.712**
sav snacks	cakes	-0.026	-0.014	-0.112	-0.005	-0.011	0.012	-0.217	-0.002	-0.130
meat	cakes	-0.009	-0.037	-0.031	-0.003	-0.079	0.004	0.012	-0.030	0.087
dairy	cakes	0.031	-0.067	-0.012	-0.046	-0.035	-0.005	0.005	0.053	0.066
fruit and veg	cakes	-0.082	-0.052	0.062	-0.063	-0.095	-0.201	-0.104	-0.130	-0.189
other	cakes	0.014	-0.002	-0.057	0.077	0.019	-0.081	0.060	-0.016	-0.054
sugary drink	sav snacks	0.305	0.161	0.158	0.364	0.558	0.323	-0.219	-0.029	0.606
med-sug drink	sav snacks	-0.060	-0.285	1.006	-1.211	-0.622	0.138	-0.215	-0.757	-1.500
low-sug drink	sav snacks	0.128	-0.076	0.208	0.182	-0.180	-0.016	-0.164	0.021	0.155
other drink	sav snacks	-0.023	0.077	0.067	0.143	0.108	0.060	0.199	-0.102	0.318
alcohol	sav snacks	0.200	0.023	0.145	0.147	0.133	-0.159	0.177	0.359	-0.159
biscuits	sav snacks	0.226	-0.068	0.054	0.158	0.342	0.152	0.125	0.134	0.225
confec	sav snacks	0.437	0.076	0.343	0.133	0.462	0.141	-0.005	0.203	0.950
cakes	sav snacks	0.321	0.172	0.775	0.034	0.399	0.617	0.335	0.270	0.957
sav snacks	sav snacks	**-0.682**	**-0.743**	**-0.873**	**-0.604**	**-0.781**	**-0.685**	**-0.741**	**-0.673**	**-0.871**
meat	sav snacks	0.083	0.019	-0.168	0.011	-0.084	0.121	0.062	-0.069	-0.412
dairy	sav snacks	-0.037	0.012	0.020	-0.114	0.009	-0.007	0.016	-0.092	-0.097

fruit and veg	sav snacks	0.034	0.114	0.076	0.148	0.005	-0.050	0.215	0.043	-0.157
other	sav snacks	-0.255	-0.096	-0.179	-0.146	-0.148	-0.068	-0.178	-0.135	0.115
sugary drink	meat	0.113	-0.234	-0.614	-0.052	-0.435	-0.660	-0.168	-0.283	-0.894
med-sug drink	meat	1.469	1.062	0.092	0.937	2.122	1.154	-0.191	0.865	0.975
low-sug drink	meat	-0.167	0.119	-0.144	0.022	0.167	-0.385	0.201	-0.011	-0.176
other drink	meat	-0.392	-0.046	-0.342	-0.340	-0.329	-0.680	0.087	-0.110	0.745
alcohol	meat	0.227	0.144	0.083	0.132	-0.025	0.241	0.069	-0.134	-0.039
biscuits	meat	-0.289	-0.113	-0.196	-0.121	-0.331	-0.457	0.034	-0.176	-0.300
confec	meat	0.421	0.598	0.563	0.636	0.154	0.062	0.538	0.424	0.610
cakes	meat	-0.209	-0.349	-0.073	-0.439	-0.170	-0.209	-0.339	-0.269	-1.070
sav snacks	meat	-0.294	-0.178	-0.032	0.080	0.125	-0.041	0.059	0.000	0.133
meat	meat	**-0.781**	**-0.842**	**-0.732**	**-0.733**	**-0.693**	**-0.757**	**-0.838**	**-0.691**	**-0.447**
dairy	meat	-0.032	-0.048	0.063	-0.119	-0.012	-0.028	-0.058	0.060	0.029
fruit and veg	meat	0.044	-0.060	-0.126	-0.098	-0.177	-0.013	-0.138	-0.138	0.032
other	meat	-0.189	-0.123	-0.061	-0.138	-0.069	-0.044	-0.077	-0.058	-0.241
sugary drink	dairy	0.396	0.297	0.738	0.502	0.001	0.244	0.224	0.426	0.080
med-sug drink	dairy	0.028	0.327	0.522	-0.743	-0.745	0.235	-0.779	-0.220	-0.104
low-sug drink	dairy	0.315	0.196	0.060	0.040	0.152	0.054	0.227	0.119	0.215
other drink	dairy	0.250	0.141	0.107	0.136	0.095	-0.029	0.214	0.196	-0.192
alcohol	dairy	0.093	-0.076	0.229	0.031	-0.022	-0.228	0.333	-0.008	-0.289
biscuits	dairy	-0.022	0.005	-0.005	0.018	-0.100	0.032	-0.040	-0.160	0.361
confec	dairy	-0.208	-0.386	-0.316	-0.398	-0.292	-0.076	-0.168	-0.205	-0.407
cakes	dairy	0.042	-0.198	0.330	0.049	-0.097	0.256	0.101	0.237	0.290
sav snacks	dairy	-0.104	0.068	0.175	-0.005	-0.018	0.160	0.084	-0.111	-0.149
meat	dairy	-0.202	-0.170	-0.062	-0.122	-0.104	-0.098	-0.164	-0.081	0.018
dairy	dairy	**-0.881**	**-0.871**	**-1.052**	**-0.906**	**-0.887**	**-0.880**	**-0.805**	**-0.923**	**-0.752**
fruit and veg	dairy	-0.049	0.046	0.011	0.061	0.023	0.029	-0.097	-0.030	0.039
other	dairy	-0.039	0.008	-0.134	-0.003	0.052	0.037	-0.118	0.005	0.015
sugary drink	fruit and veg	0.093	-0.078	0.167	-0.319	-0.132	-0.197	0.177	-0.145	-0.372
med-sug drink	fruit and veg	0.366	0.349	0.050	0.414	0.384	0.388	1.088	0.344	0.833
low-sug drink	fruit and veg	0.232	-0.037	0.257	0.120	0.194	0.102	0.268	0.108	-0.020
other drink	fruit and veg	0.298	-0.082	-0.151	0.132	-0.063	0.008	0.281	0.179	-0.239
alcohol	fruit and veg	-0.148	-0.101	-0.104	-0.100	-0.128	0.135	-0.069	-0.092	0.100
biscuits	fruit and veg	-0.049	0.075	0.053	0.201	0.162	-0.175	-0.009	-0.315	-0.267
confec	fruit and veg	-0.220	-0.382	-0.195	-0.504	-0.044	-0.011	-0.323	-0.081	-0.430
cakes	fruit and veg	0.035	-0.124	0.030	0.124	-0.127	0.142	-0.183	-0.392	0.035
sav snacks	fruit and veg	0.019	0.130	0.217	0.028	0.023	0.072	0.131	-0.139	0.073
meat	fruit and veg	-0.132	-0.067	-0.006	-0.151	-0.090	-0.163	-0.083	-0.166	0.013
dairy	fruit and veg	-0.151	-0.123	-0.189	-0.108	-0.116	-0.066	-0.130	-0.119	0.107
fruit and veg	fruit and veg	**-0.605**	**-0.573**	**-0.693**	**-0.561**	**-0.595**	**-0.699**	**-0.618**	**-0.574**	**-0.568**
other	fruit and veg	-0.010	-0.020	-0.047	0.007	-0.038	-0.092	-0.087	0.033	-0.154
sugary drink	other	-0.730	0.015	-0.346	0.277	-0.229	0.420	0.039	0.255	0.558
med-sug drink	other	-0.267	0.151	0.340	0.638	-0.628	-1.152	0.473	0.412	0.733
low-sug drink	other	0.111	0.063	-0.115	-0.147	-0.137	0.512	-0.284	0.024	-0.006
other drink	other	-0.290	-0.146	0.101	0.068	-0.067	0.827	-0.370	0.528	0.064
alcohol	other	-0.409	-0.303	-0.394	-0.455	-0.232	-0.422	-0.729	-0.420	-0.314
biscuits	other	-0.307	-0.285	-0.275	-0.464	-0.756	-0.144	-0.395	-0.365	-0.359
confec	other	-0.821	-0.677	-0.860	-0.467	-1.202	-1.194	-1.211	-1.190	-1.032
cakes	other	-0.636	-0.298	-1.402	-0.544	-0.450	-0.851	-0.465	-0.599	-1.233
sav snacks	other	0.189	-0.313	-0.299	-0.360	-0.347	-0.196	-0.474	0.042	-0.195
meat	other	0.033	0.064	-0.025	-0.212	0.074	-0.046	-0.230	-0.036	0.131
dairy	other	-0.137	-0.094	-0.100	-0.084	-0.070	-0.300	0.052	-0.109	-0.511
fruit and veg	other	-0.229	-0.161	0.096	-0.273	0.050	0.014	0.151	0.021	0.232
other	other	**-0.499**	**-0.680**	**-0.643**	**-0.501**	**-0.641**	**-0.641**	**-0.450**	**-0.697**	**-0.712**

*Estimated from Kantar FMCG panel data 2012-2013; unweighted sample estimates

3. Modelling Equations

A. Energy purchase

Average baseline daily purchases of energy were estimated using 2013 UK Kantar FMCG panel data (n=32,620 households) and calculated per household member per day. No adjustment for age or gender was applied as this information was available for the "main shopper" but not for other household members. Baseline purchases were expressed in weighted means to account for sampling selection of the panel and under-reporting using gross-up weights provided by Kantar.

Analyses were carried out on the full sample and subsamples by household annual income (low income (<£20,000), middle income (£20,000–£49,999) and high income (≥£50,000)) and Body Mass Index ("not overweight" (BMI <25kg/m^2), "overweight" (BMI 25 to <30 kg/m^2), and "obese" (BMI ≥30 kg/m^2)) of the main household purchaser [i.e. the person generally responsible for food purchase in a household].

The following sample sizes were used for the core analysis:

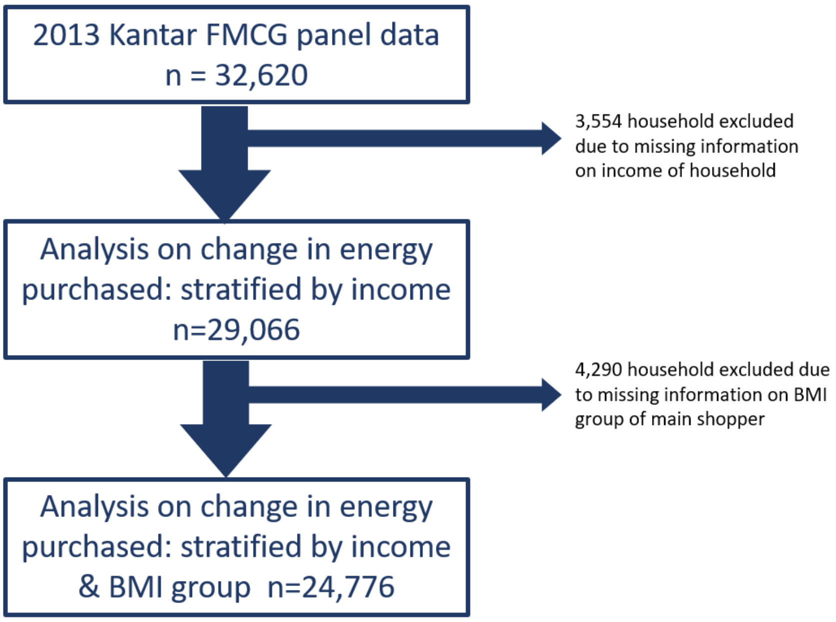

Sensitivity analysis was performed removing the households with children from the database. Analysis stratified by income was therefore performed on 15,862 adult-only households.

We used the own- and cross-PED matrix for all BMI/income subgroups to estimate change in per person energy purchase simulating a 20% price increase on one or multiple sweet snack food groups. New energy purchase totals per income and/or BMI group were constructed by totalling scenario specific energy purchases per food group - running 1000 MC simulations for each sub-group. See equation 1.

[Equation 1]: "New" estimate of energy purchased based on a 20% increase in price of high-sugar snacks

$$\mathrm{EP}_{\mathrm{all}_{\mathrm{new}}}(\mu,\beta) = \sum_{f=1}^{13} \left(t_{f(i)} * p_{f(i)}(\mu, var)\, EP_{f(i)}(\mu,\beta) + c_{f(j(i))}\, EP_{f(j(i))}(\mu,\beta) + c_{f(k(i))} EP_{f(k(i))}(\mu,\beta) \ldots + \ldots c_{f(n(i))} EP_{f(n(i))}(\mu,\beta) \right) \quad \textbf{[1]}$$

Where:

$EP_{all\_new(x)}$ = Energy Purchased based on all food groups combined under price increase scenario (x)

f(i) = food group (1-13);

t = price increase (%);
p=own-PED;
c=cross-PED;
i = food group with price change;
j, k and n = other food groups/potential substitutes;
μ = mean energy purchased;
β= uncertainty estimate
var = variance of estimate

Confidence intervals of changes in energy purchase were constructed using a probabilistic distribution of intra (BMI and income) group variability of energy purchased, as well as the probabilistic distribution of the uncertainties around own PEDs and cross-PEDs estimates, assuming additive uncertainty.

To estimate the difference in energy purchased under price increase scenarios as compared to baseline levels of energy purchased – running 1000 MC simulation for each sub-group (Equation 2).

[Equation 2]: Difference in energy purchase based on a 20% increase in price of high-sugar snacks

$$\Delta \mathrm{EP}_{\mathrm{all,t(i)}} \approx \mathrm{EP}_{\mathrm{all\_original}}(\mu, \beta) - \mathrm{EP}_{\mathrm{all\_new,t(i)}}(\mu, \beta) \qquad \textbf{[2]}$$

Where
ΔEP_{all} = Change in energy purchase of all foods combined;
t=prince increase scenario;
i = specific food group of price increase (biscuits, cake, confectionary, combination);
μ = mean energy purchased;
β= uncertainty estimate;
$EP_{all\_original}$= Original energy purchased before price changes – as reported in Kantar FMCG data

B. Weight loss

We used a static model to estimate average weight change for each BMI and income group based on change in energy intake. We used an average of 7715kcal per kg weight loss, with a standard deviation of 245 kcal (representing a 7% coefficient of variation (CV) and assumed a normal distribution. We disregarded any- changes in energy expenditure resulting from weight fluctuations, as we expected the change in weight and hence energy expenditure to be small over the course of one year. For each sub-group 1000 Monte Carlo simulations were run using best estimates and uncertainty of change in energy purchase and variance of energy expenditure.

[Equation 3]: Weight loss estimations based on change in energy purchase

$$\mathrm{W(t)} = W_0 + \frac{\Delta EP_{all\left(t_{(i\ldots n)}\right)}(\mu,\beta)}{\theta\ (\mu,var)} \qquad \textbf{[3]}$$

Where
W=body weight;
EP= energy purchase;
t=price increase scenario (%);
θ = energy expenditure for 1 kg of body weight loss
μ = mean energy purchased;
β= uncertainty estimate
var = variance of estimate

4. Approach to uncertainty

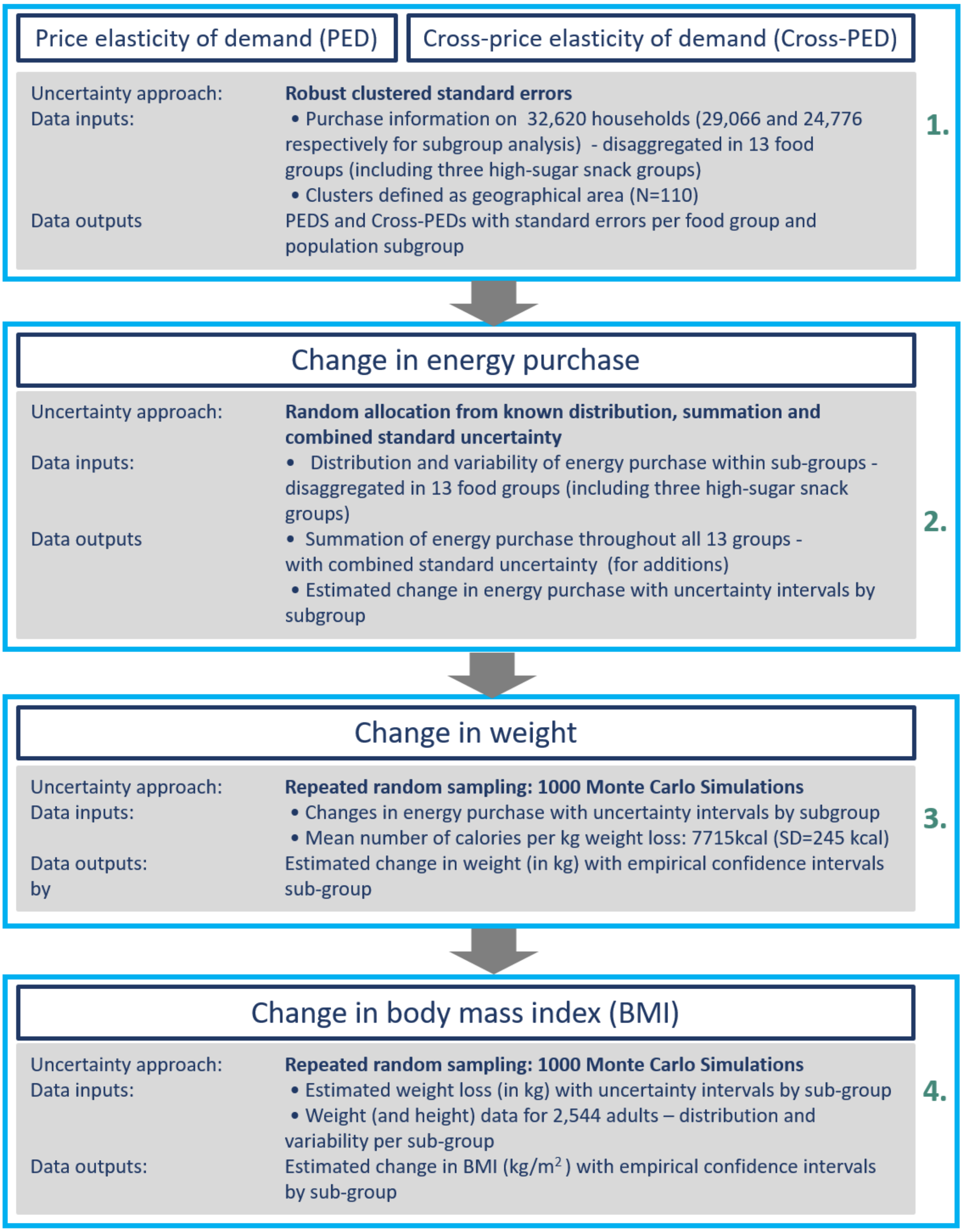

Supplement: Supplementary file 2 — Supplementary information: technical appendix [file schp048338.ww2.pdf]
